# Ultrasound high-definition microvasculature imaging with novel quantitative biomarkers improves breast cancer detection accuracy

**DOI:** 10.1007/s00330-022-08815-2

**Published:** 2022-04-29

**Authors:** Redouane Ternifi, Yinong Wang, Juanjuan Gu, Eric C. Polley, Jodi M. Carter, Sandhya Pruthi, Judy C. Boughey, Robert T. Fazzio, Mostafa Fatemi, Azra Alizad

**Affiliations:** 1 Department of Physiology and Biomedical Engineering, Mayo Clinic College of Medicine and Science, Rochester, MN USA; 2 Department of Radiology, Mayo Clinic College of Medicine and Science, 200 1st Street SW, Rochester, MN 55905 USA; 3 Department of Health Science, Mayo Clinic College of Medicine and Science, Rochester, MN USA; 4 Department of Laboratory Medicine and Pathology, Mayo Clinic College of Medicine and Science, Rochester, MN USA; 5 Department of Medicine, Mayo Clinic College of Medicine, Rochester, MN USA; 6 Department of Surgery, Mayo Clinic College of Medicine and Science, Rochester, MN USA

**Keywords:** Breast cancer, Contrast-agent-free method, Neovascularization, Quantitative biomarkers, Ultrasound

## Abstract

**Objectives:**

To overcome the limitations of power Doppler in imaging angiogenesis, we sought to develop and investigate new quantitative biomarkers of a contrast-free ultrasound microvasculature imaging technique for differentiation of benign from malignant pathologies of breast lesion*.*

**Methods:**

In this prospective study, a new high-definition microvasculature imaging (HDMI) was tested on 521 patients with 527 ultrasound-identified suspicious breast masses indicated for biopsy. Four new morphological features of tumor microvessels, microvessel fractal dimension (mvFD), Murray’s deviation (MD), bifurcation angle (BA), and spatial vascularity pattern (SVP) as well as initial biomarkers were extracted and analyzed, and the results correlated with pathology. Multivariable logistic regression analysis was used to study the performance of different prediction models, initial biomarkers, new biomarkers, and combined new and initial biomarkers in differentiating benign from malignant lesions.

**Results:**

The new HDMI biomarkers, mvFD, BA, MD, and SVP, were statistically significantly different in malignant and benign lesions, regardless of tumor size. Sensitivity and specificity of the new biomarkers in lesions > 20 mm were 95.6% and 100%, respectively. Combining the new and initial biomarkers together showed an AUC, sensitivity, and specificity of 97% (95% CI: 95–98%), 93.8%, and 89.2%, respectively, for all lesions regardless of mass size. The classification was further improved by adding the Breast Imaging Reporting and Data System (BI-RADS) score to the prediction model, showing an AUC, sensitivity, and specificity of 97% (95% CI: 95–98%), 93.8%, and 89.2%, respectively.

**Conclusion:**

The addition of new quantitative HDMI biomarkers significantly improved the accuracy in breast lesion characterization when used as a complementary imaging tool to the conventional ultrasound.

**Key Points:**

*• Novel quantitative biomarkers extracted from tumor microvessel images increase the sensitivity and specificity in discriminating malignant from benign breast masses.*

*• New HDMI biomarkers Murray’s deviation, bifurcation angles, microvessel fractal dimension, and spatial vascularity pattern outperformed the initial biomarkers.*

*• The addition of BI-RADS scores based on US descriptors to the multivariable analysis using all biomarkers remarkably increased the sensitivity, specificity, and AUC in all size groups.*

**Supplementary Information:**

The online version contains supplementary material available at 10.1007/s00330-022-08815-2.

## Introduction

Invasive breast carcinoma is an angiogenesis-dependent malignancy, and studies have indicated that an increased tumor microvessel density is associated with poor prognosis [1, 2]. Importantly, blood vessels in malignant tumors are extremely heterogeneous and very different from vessels found in normal tissues or benign tumors. Poor oxygen levels in early-emerging tumors stimulate the release of vascular endothelial growth factors (VEGF), which initiates new vascularization and tumor growth [2, 3]. In turn, the demand for additional oxygen in growing tumors leads to formation of leaky, fragile, tortuous vessels [4]. In contrast, in most benign cases, tumor growth is controlled by mechanisms similar to those of normal tissue, leading to the creation of organized and non-tortuous vessel shapes [4, 5].

Conventional Doppler methods with differentiating potential in breast masses [6–10] are sensitive only to fast flows, leading to highly fragmented and patchy images of the underlying vessels, preventing structural analysis of microvessels. The utility of photoacoustic imaging approaches has been shown for microvessel architectural differences in superficial breast lesions [11], but has limited use in deep-seated tumors. Contrast-enhanced ultrasound (US) has been investigated for increasing the specificity of ultrasound for differentiation of benign and malignant breast masses [12, 13]. Acoustic angiography and ultrasound localization microscopy [14–16], with the help of contrast agents, could resolve microvessels in preclinical studies [17].

Recently, obtaining fine vascular features of breast tumors at super-resolution scales was possible in a spontaneous mouse model of breast cancer [18] and in humans [19], but this approach is associated with inconvenience and increased cost associated with injection of contrast agents. Contrast-free ultrasound imaging of tumor microvessels for differentiation of malignant from benign breast masses has been investigated; however, these efforts were limited to a pixel count method and visual inspection of images for the assessment of vessel shapes and distribution [20–22]. To address these research gaps, we have previously developed a contrast-free ultrasound-based technology to visualize small submillimeter vessels (as small as 300 μm) and quantify tumor microvessel morphological structures, named quantitative high-definition microvasculature imaging (qHDMI) [23, 24]. The objective of this research is to complement the gray scale morphology–based assessment of conventional ultrasound with the microvasculature features of breast tumor for increased accuracy in cancer detection.

Recently, the basic principles of 4 new quantitative biomarkers based on microvessel images, as well as some simulations and limited patient study results, were presented to illustrate the role of each biomarker [25]. The four biomarkers are (1) microvessel fractal dimension, (2) Murray’s deviation, (3) bifurcation angle (BA), and (4) spatial vascular pattern [25]. The goal of this study is to investigate the performance of the four newly developed HDMI quantitative biomarkers on a relatively large population. Thus, more lesion categories allowed us to investigate the performance of HDMI individual biomarkers and the combination of them in a multivariable analysis for different pathologies and different lesion size groups. The study also tests the performance of multiple prediction models, using only new biomarkers, only initial biomarkers, and a combination of new and initial with or with or without Breast Imaging Reporting and Data System (BI-RADS) scores. Furthermore, the correlation of HDMI biomarkers with cancer grades has been investigated. As such, the current validation study substantially expands the previous works. The proposed method objectively classifies the tumor as benign or malignant, which makes this method operator independent and eliminates the observer/reader variability for a reliable clinical use.

## Materials and methods

### Participants

**Fig. 1:**
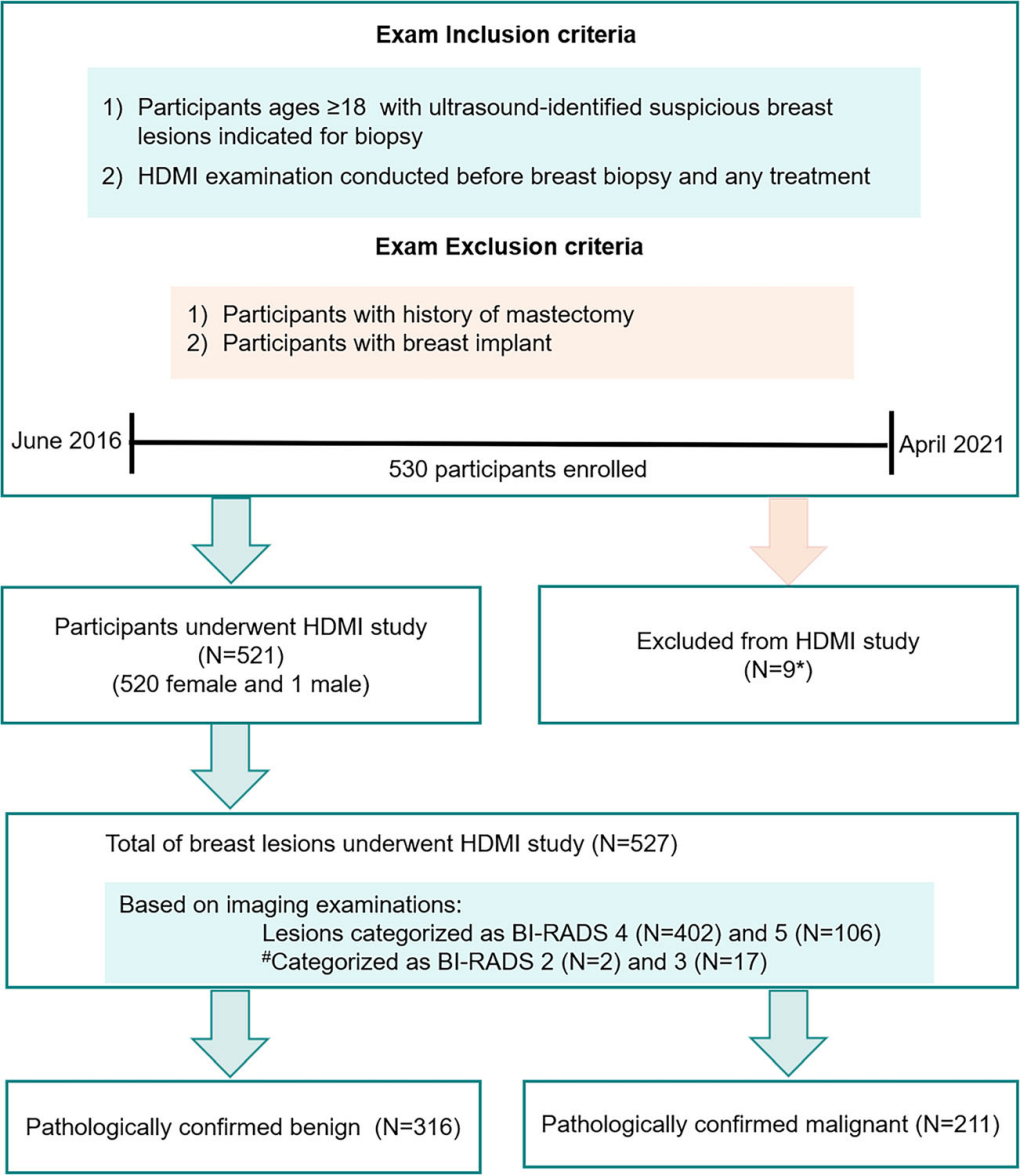
Flowchart for the study participants. *Nine patients were excluded from HDMI study because the lesions were lymph nodes. #BI-RADS 2 and 3 were biopsied due to patient preference. HDMI = high-definition microvasculature imaging

We received institutional review board approval in compliance with the Health Insurance Portability and Accountability Act. A signed written informed consent with permission for publication was obtained from each enrolled participant prior to the prospective study. Patients were prospectively enrolled at the Department of Radiology, Breast imaging Division. From June 2016 to April 2021, 530 patients with ultrasound-identified suspicious breast masses indicated for biopsy were consecutivelyenrolled for the study. As expected, most cases were classified as BI-RADS scores 4 and 5; those patients with BI-RADS 2 and 3 included in this study all underwent biopsy because of the risk factors such as the history of breast cancer in a first-degree family member and the will of the patient for biopsy. Details of participant selection, inclusion and exclusion criteria, are provided in Fig. 1. Lesions were assigned BI-RADS assessments by different radiologists, and the investigative team was blinded to these assessments during the investigation. HDMI results were not used for the clinical decision for the enrolled patients. In total, 521 participants with 527 lesions were included in this HDMI study. Demographic characteristics are shown in Table 2. After HDMI research examination, all patients underwent core needle biopsy within an hour, from which histopathological results served as the gold diagnostic standard. The results of HDMI were not available to the pathologist who assessed and reported the breast biopsy. The pathology results of core-needle biopsy rather than the surgical pathology served as the gold reference standard because of the following: (1) surgical pathology is not available in benign lesions as benign lesions do not normally have surgical excision for treatment and (2) there is no cancer for patients who are complete pathological responders to neoadjuvant therapy.

### High-definition microvasculature imaging and quantitative biomarkers

The ultrasound examinations were performed by two sonographers with more than 30 and 15 years of US scanning experience, respectively. The sonographers were instructed to minimize the preload to reduce unwanted pressure on the tissue microvessels. To reduce motion artifacts, patients were instructed stay still and suspend respiration for approximately 3 s during data acquisition. To increase reproducibility, 2 acquisitions at each scan orientation were acquired. For each participant, only one of the two sonographers conducted the HDMI scanning.

Using an ultrasound platform with capability of plain wave imaging (Alpinion Ecube12-R, ALPINION Medical Systems), and a linear array L3-12H operating at 8.5 MHz, breast lesions were identified on plane-wave B-mode. Then a sequence of high frame rate data (at ~ 600 frames per second) was acquired on the lesion site. This ultrasound system provides a sequence of frames in the form of raw in-phase and quadrature beamformed data for a total duration of 3 s. Each frame of the data was formed using 5-angle coherent plane-wave compounding [26]. The methods for obtaining HDMI images, vessel extraction, and steps for vessel segmentation [23, 24] have been detailed in the supplemental material, available online.

Definitions and calculations of the new biomarkers (1) microvessel fractal dimension (mvFD) [25, 27, 28], (2) bifurcation angle (BA) [25, 29, 30], (3) Murray’s deviation (MD) [25, 31, 32], and (4) spatial vascularity pattern (SVP) [25, 33] calculated by vessel density ratio (VDR) [34] as well as initial biomarkers [24] are detailed in Table 1 and Supplementary material.

**Table 1:** HDMI quantitative biomarkers

Biomarkers	Definition and calculation
Initial HDMI biomarkers	
NB	Numbers of branch points: defined as any node that connected to three or more vessel segments [24]
NV	Number of vessel segments
VD	Vessel density: defined as the proportion of vessel area with blood flow over the total area measured [24]
*D* (mm)	Vessel diameter (*D*_mean_, *D*_max_): defined as two times of the minimum distance between the vessel centerline and the vessel border [24]
DM	Distance metric (DM_mean_, DM_max_): defined as the ratio between the actual path length of a meandering curve (vessel) and the linear distance between the two end points [24]. Distance metric measures vascular tortuosity
Novel HDMI biomarkers	
MD	Murray’s deviation: diameter mismatch, defined as the deviation from Murray’s law, increases in the vasculature network of malignant tumors Using skeleton image, the diameters of sub-vessels were used to define the mother vessel (the sub-vessel with the largest diameter) and daughter vessels (the remaining sub-vessels) MD was calculated using $\mathrm{MD}=\frac{\left|{D}_{\mathrm{mother}}^3-\sum {D}_{\mathrm{daughter}}^3\right|}{D_{\mathrm{mother}}^3}$. If NB = 0, MD = 1 [25, 31, 32]
mvFD	Microvessel fractal dimension: A unit-less geometrical feature is a marker of microvascular complexity. Can be calculated using the box counting method. Knowing the box size, *s*, and the number of boxes, *N*_*s*_, to cover all the vessels, the mvFD can be calculated,$\mathrm{mvFD}=\underset{s\to 0}{\lim}\frac{\log {N}_s}{\log \frac{1}{s}}$, to identify the structural complexity of tumor vessels [25, 27, 28]
BA (°)	Bifurcation angle: refers to the angle between two daughter vessels Two straight lines were generated by fitting two daughter vessels, and the angle between them is calculated as BA [25, 29, 30] If NB = 0, BA = 180°
VDR	Vessel density ratio (VDR): tumor vessel distributions at the periphery (VDR < 1), or at the center (VDR > 1) or both (VDR ≈ 1)$\mathrm{VDR}=\frac{{\mathrm{Vessel}\ \mathrm{Density}}_{\mathrm{center}}}{{\mathrm{Vessel}\ \mathrm{Density}}_{\mathrm{peripheral}}}$ [34]
SVP	Spatial vascularity pattern: the distribution pattern of microvessels, either concentrated peripherally (peritumoral vascularization) or inside the lesion (intratumoral vascularization). SVP is calculated by VDR. If VDR < 1, SVP = 0, meaning a more peripherally concentrated vessel distribution. If VDR > 1, SVP = 1, meaning a more centrally concentrated vessel distribution [25, 33]

*HDMI* high-definition microvasculature imaging

### Clinical pathologic data (please see supplemental materials)

#### Data analysis

For each image, a statistical distribution of the new and initial HDMI biomarkers was obtained. Using pathology results as the gold standard, vessel morphological features were tested for statistical significance in differentiating between benign and malignant lesions using receiver operating characteristics (ROC) analysis. For each new biomarker, error-bar plots with 95% confidence intervals (CI) were obtained for different lesion size constraints. Specificity, sensitivity, area under the curve (AUC), and 95% CI were obtained. Statistical significance analyses were performed using a Wilcoxon rank-sum test using R (version 3.6.2), with a *p* value < 0.05 considered significant. The correlation between two biomarkers was calculated using the cor function with the Pearson method. In addition to analyzing the performance of individual biomarkers, a multivariable logistic regression analysis was done to study the performance of the combination of all new and initial HDMI biomarkers in differencing lesions. Further, BI-RADS with US descriptors [35, 36], used to categorize breast lesions to select candidates for biopsy, were included in our analysis to determine the added value of quantitative HDMI for increased detection accuracy. The malignancy probability was calculated with the following equation: $\mathrm{probability}=\mathrm{logi}{\mathrm{t}}^{-1}\left(B+\sum \limits_{i=1}^m{C}_m{P}_m\right)$, where *B* is a constant obtained from the multivariable logistic regression analysis; *P*_*m*_ is the quantitative HDMI biomarker, or the BI-RADS score, and *C*_*m*_ is the coefficient for the corresponding quantitative biomarker obtained from the multivariable logistic regression analysis; *m* is the number of quantitative biomarkers included in the prediction model, and the logistic function logit^−1^ is defined as logit^−1^(*α*) = 1/(1 + exp(−*α*)).

## Results

Of a total of 527 breast lesions examined by HDMI, 316 were benign and 211 were malignant. Table 2 shows the participant demographic and lesion characteristics. The distribution of lesion types by pathology are summarized in Table 3. The most common benign histologic type was fibroadenoma. As expected, invasive primary breast carcinoma comprised most of the malignant tumors, with 66% as invasive ductal carcinoma (IDC). The Nottingham grades of each invasive breast cancer type are provided in Table 3.

**Table 2:** Demographic and lesion characteristics

	Benign	Malignant	Statistical significance (*p* value)
Gender (F/M)	316/0	211/1	NA
Age (y)	49 ± 15 (18–88)	61 ± 12 (27–89)	< 0.0001
Mass size (mm)	14 ± 8.6 (4–60)	18.5 ± 11.9 (5–72)	< 0.0001
BI-RADS			< 0.0001
2	2	0	
3	17	0	
4	287	115	
5	10	96	

Mean values of age and tumor size are shown with standard deviations and minimum–maximum intervals in parentheses. Statistical comparison of age, mass size, and BI-RADS between benign and malignant groups was performed using a Wilcoxon rank sum test (last column); a *p* value < 0.05 was considered to reflect statistical significance

Non applicable

**Table 3:** Distribution of lesion types by pathology in a total of 527 breast lesions

Breast lesions	Lesion number	Percentage
Total breast lesions	527	NA
Total benign lesions	316	60% (316/527)
Fibroadenoma	114	36% (114/316)
Benign changes/stromal fibrosis	77	24% (77/316)
Fibrocystic changes	31	10% (31/316)
Papilloma	28	9% (9/316)
Pseudoangiomatous stromal hyperplasia	22	7% (22/316)
Fat necrosis	18	6% (6/316)
Atypia^a^	14	4% (14/316)
Duct ectasia	6	2% (6/316)
Adenosis	4	1% (4/316)
Others^b^	2	1% (2/316)
Total malignant lesions	211	40% (211/527)
Primary breast carcinomas	209	99% (209/211)
Invasive ductal carcinoma	138	66% (138/209)
Invasive mammary carcinoma with mixed ductal and lobular features	30	14% (30/209)
Invasive lobular carcinoma	26	12% (26/209)
Ductal carcinoma in situ	15	7% (15/209)
Non-mammary malignancies in breast	2	1% (2/211)
Malignant grade for invasive breast carcinomas	194	
Grade I	50	26% (50/194)
Grade II	92	47% (92/194)
Grade III	52	27% (52/194)
Malignant grade for ductal carcinoma in situ	15	
Low grade	7	46% (7/15)
Intermediate grade	4	27% (4/15)
High grade	4	27% (4/15)

Numbers in parentheses represent the numerator and denominator for the corresponding percentage

^a^Atypia: 8 atypical ductal hyperplasia, 2 atypical lobular hyperplasia, 2 atypical papillary lesion, 1 radial scar with focal residual atypical hyperplasia associated with flat epithelial atypia, and 1 atypical/high-risk and fibrocystic changes

^b^Others: 1 ductal hyperplasia and 1 organizing abscess with associated granulomatous reaction

Non applicable

**Fig. 2:**
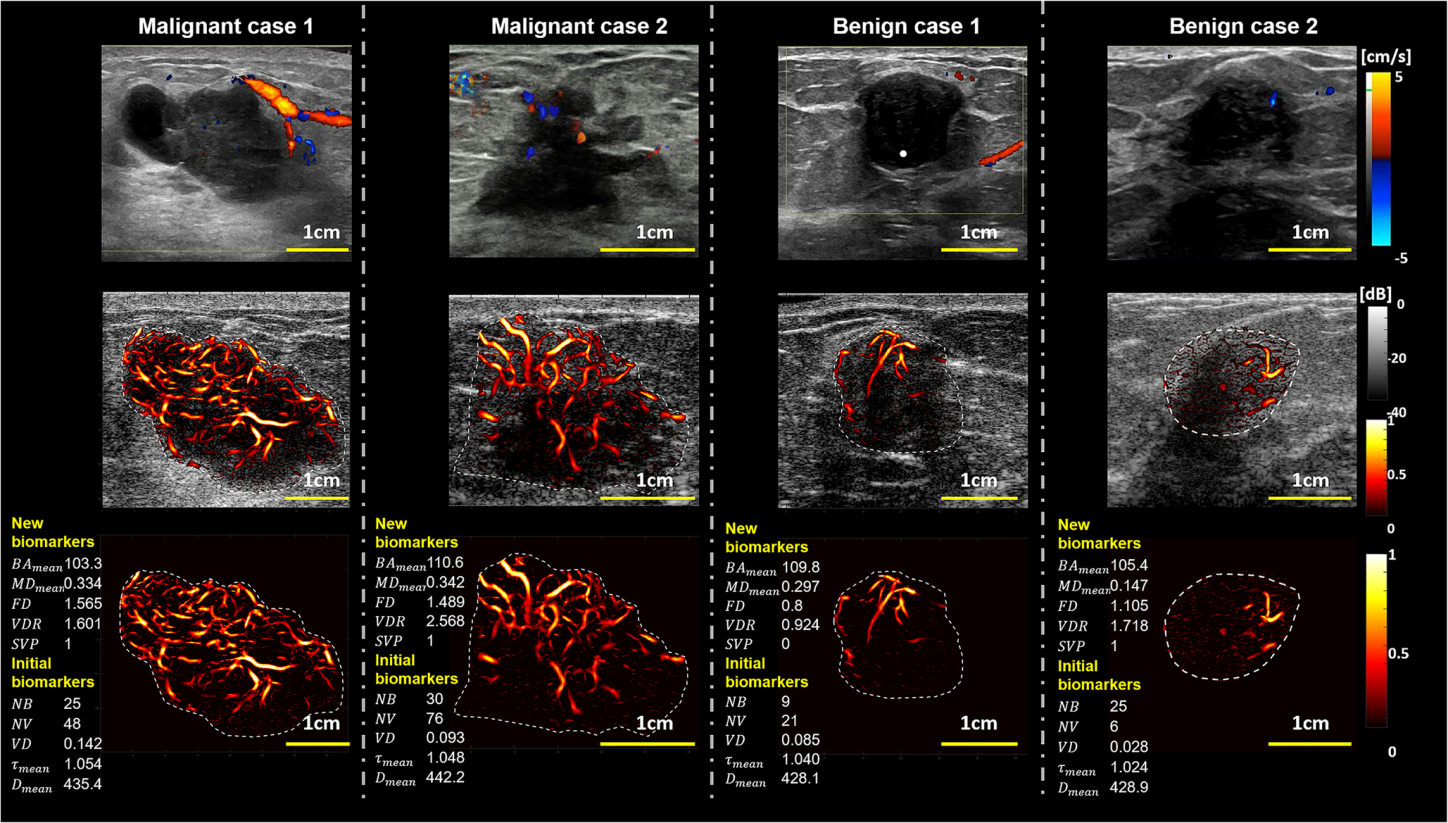
Representative cases of tumor vasculature images using different imaging methods. Regular color Doppler (1^st^ row), overlay HDMI on B-mode (2^nd^ row), and the HDMI of the breast mass (3^rd^ row). The representative new and initial biomarkers are shown at the left side for each HDMI image (3rd row). The two columns on the left represent two malignant breast masses (M1 and M2), and the two columns on the right represent benign breast lesions (B1 and B2). The histological results for both malignant cases (M1 and M2) are reported as invasive mammary carcinoma with mixed ductal and lobular features, grade III, and invasive ductal carcinoma grade III, respectively. The biopsy results for the benign cases (B1 and B2) are indicated as benign fibroadenoma. The reference clinical Doppler images shown on row 1 were acquired in a clinical setting by a different sonographer using a clinical ultrasound scanner different from the research ultrasound platform used by the investigative team

Figure 2 is a visual presentation of the HDMI images of malignant and benign breast lesions in comparison with conventional Doppler. While conventional Doppler images showed slightly higher blood activity in the form of patchy and large vessels in the malignant cases, HDMI provided high-definition images of both increased peripheral and internal microvascularity with higher complexity than seen in benign masses with noticeably fewer microvessels and less complex morphology. This increased sensitivity and enhanced image resolution of HDMI enabled additional quantitative analysis of vessel morphological features by extracting vessel skeleton and branching into vessel segments, leading to a classification power of HDMI biomarkers as seen in the statistical results.

### Statistical results of HDMI biomarkers

**Fig. 3:**
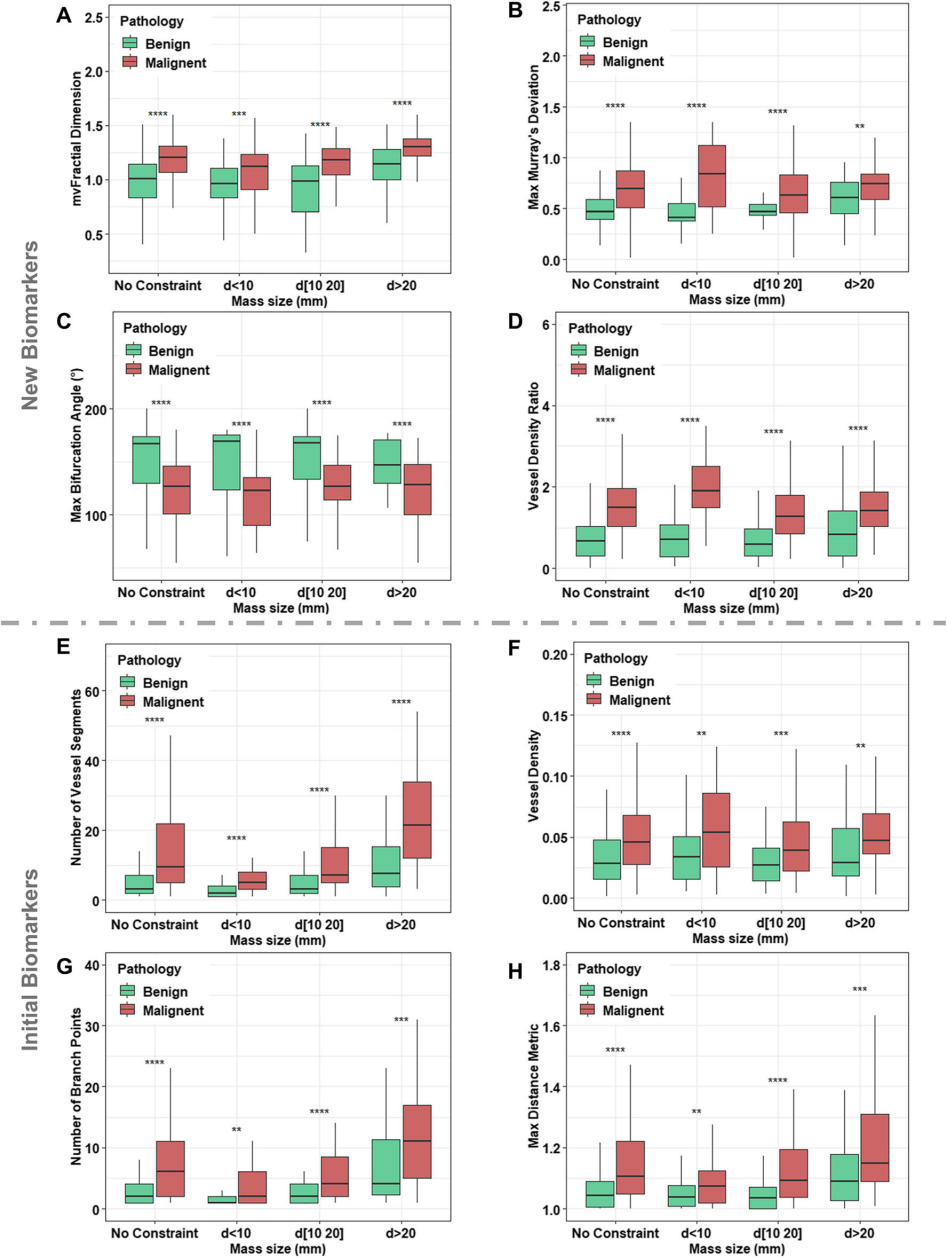
Error-bar plots for differentiation of benign and malignant breast lesions in different lesion size groups using new biomarkers: **A** mircovessel fractal dimension (mvFD), **B** maximum Murray’s deviation, **C** maximum bifurcation angle (BA), **D** vessel density ratio (VDR), and initial biomarkers: **E** number of vessel segments (NV), **F** vessel density (VD), **G** number of branch points (NB), and **H** maximum distance metric (DM_max_, tortuosity metric). *d* = lesion diameter, *p < 0.05, **p < 0.01, ***p < 0.001. No constraint: Benign (*n* = 316), Malignant (*n* = 211); *d* < 10 mm, Benign (*n* = 103), Malignant (*n* = 50); 10 ≤ *d* ≤ 20 mm, Benign (*n* = 152), Malignant (*n* = 93); *d* > 20 mm, Benign (*n* = 61), Malignant (*n* = 68)

**Fig. 4:**
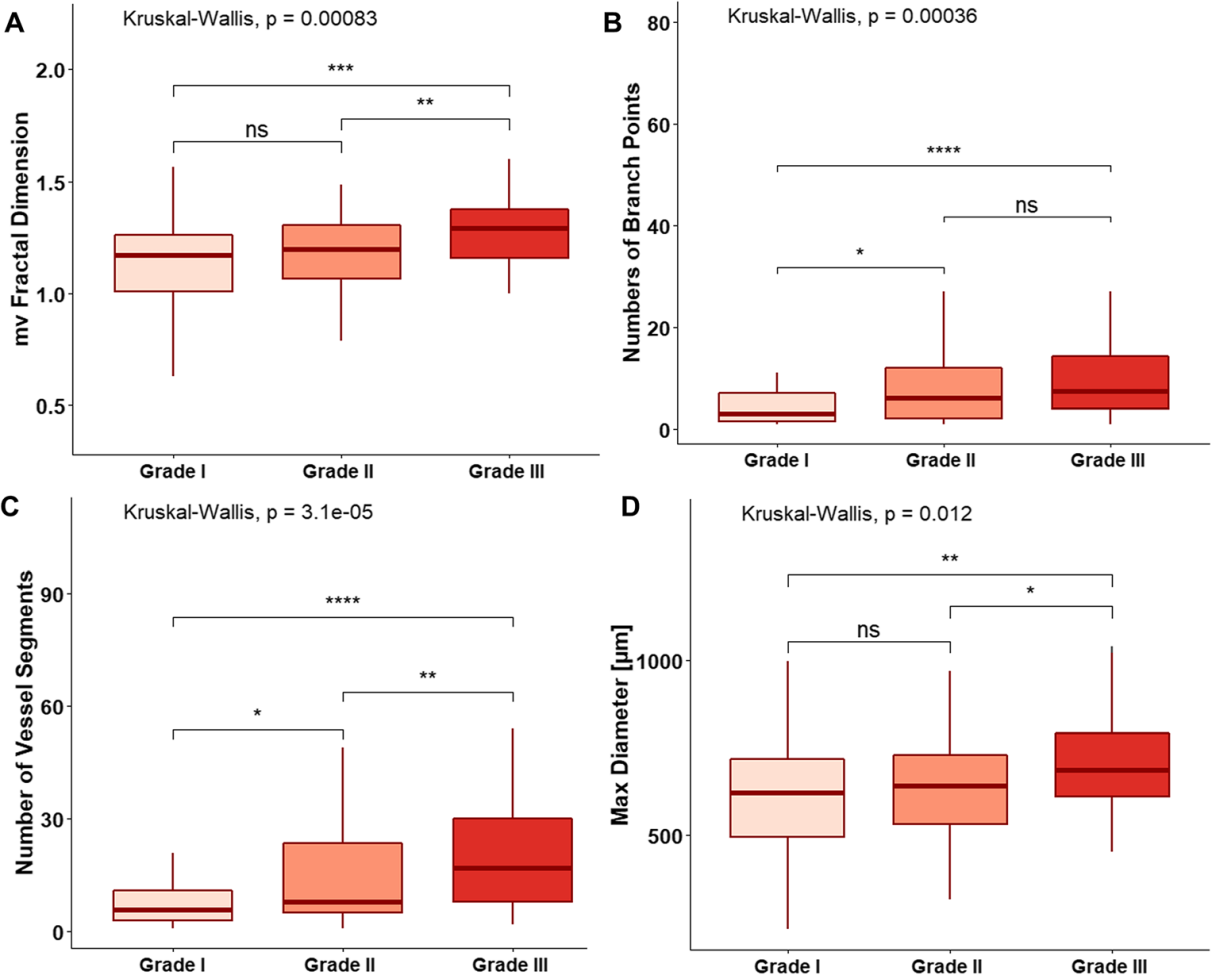
Error-bar plots of HDMI biomarkers for differentiation of invasive breast cancer grade: **A** mircovessel fractal dimension (mvFD), **B** number of branch points (NB), **C** number of vessel segments and (NV), **D** maximum diameter (*D*_max_). * *p* < 0.05, ** *p* < 0.01, *** *p* < 0.001, **** *p* < 0.0001. HDMI = high-definition microvasculature imaging

Each new HDMI biomarker shows statistically significant differences (*p* < 0.05) between malignant and benign in different size groups (Fig. 3). In the analyses of HDMI biomarkers for different pathological grades of malignant tumors, a low SVP factor was noted in grade III malignant breast tumors, which include larger-size tumors with higher vessel density in the peritumoral area. Moreover, higher values of mvFD, NV, NB, and *D*_max_ (all *p* < 0.05) were seen in IDC, NG grade III (Fig. 4). The performance of all new and initial HDMI biomarkers for lesion classification in terms of sensitivity, specificity, PPV, NPV, AUC, 95% CI, and *p* value are shown in the Supplementary table, available online.

The performance of HDMI biomarkers for predicting the status of immunohistochemical (IHC) biomarkers and that of the molecular subtypes are summarized in Table 4. Significant differences were found for FD, NV, and NB for predicting the PR status, HER2 status, and Ki-67, with PR positive presenting significantly lower FD, NV, and NB values, while HER2 positive showed significantly higher values. BA_mean_, NV, and NB showed significances in predicting the molecular subtypes. Among the five subtypes, Luminal A subtype showed the smallest NV and NB values.

**Table 4:** Diagnostic performance of HDMI biomarkers for predicting the status of ER, PR, HER2, Ki-67, and molecular subtypes in malignant masses

Immunohistochemical biomarkers	BA_mean_	^a^*p* value	FD	^a^*p* value	NV	^a^*p* value	NB	^a^*p* value
ER		0.141		0.259		0.950		0.908
Negative (25)	68.3 ± 45.2		1.2 ± 0.3		13.6 ± 12.3		6.2 ± 5.8	
Positive (159)	80.6 ± 44.1		1.1 ± 0.2		14.0 ± 14.6		6.6 ± 7.9	
PR		0.711		**0.020**		**0.019**		**0.026**
Negative (38)	80.6 ± 40.7		1.2 ± 0.3		18.6 ± 14.9		8.6 ± 7.8	
Positive (146)	78.5 ± 45.4		1.1 ± 0.2		12.7 ± 14.0		6.0 ± 7.6	
HER2		0.393		**0.036**		**0.004**		**0.016**
Negative (159)	77.1 ± 40.1		1.1 ± 0.2		12.6 ± 13.6		5.9 ± 7.3	
Positive (25)	90.6 ± 30.1		1.2 ± 0.2		22.4 ± 16.3		10.4 ± 9.2	
Ki-67		0.209		**0.036**		**0.001**		**0.004**
< 0.14 (113)	75.7 ± 46.0		1.2 ± 0.2		12.4 ± 14.2		5.8 ± 7.4	
≥ 0.14 (71)	84.1 ± 41.4		1.1 ± 0.3		16.4 ± 14.2		7.7 ± 8.0	
Subtypes		**0.047**		0.776		**0.018**		0.050
Luminal A (77)	74.3 ± 47.5		1.1 ± 0.2		11.2 ± 14.1		5.3 ± 7.5	
Luminal B (HER2+) (61)	84.5 ± 44.2		1.1 ± 0.2		14.3 ± 13.3		6.7 ± 7.4	
Luminal B (HER2−) (21)	92.4 ± 24.7		1.2 ± 0.3		23.7 ± 16.6		11.1 ± 9.6	
HER2+ (4)	81.1 ± 54.8		1.2 ± 0.2		15.8 ± 14.5		7.3 ± 6.4	
TN (21)	65.8 ± 44.3		1.2 ± 0.3		13.2 ± 12.2		5.9 ± 5.9	

Numbers in parentheses are lesion numbers

^a^A *p* value smaller than 0.05, shown in bold, indicates significance

Table 5 explains the relationship between different HDMI parameters. A correlation coefficient smaller than 0.45 indicates a low-correlation relationship (marked with * in Table 5). In other words, they are less dependent on each other; therefore, their contributions are added in predicting the diagnosis of breast cancer. Among the new biomarkers, FD has low correlation with BA_mean_ or MD_mean_. Among the initial biomarkers, *τ*_mean_ has a low correlation coefficient with other initial biomarkers. The new biomarkers BA_mean_ and MD_mean_ have low correlations with all the initial biomarkers.

**Table 5:** Summary of the correlation coefficients between the HDMI biomarkers

	BA_mean_	MD_mean_	FD	*τ*	*D*_mean_	NB	NV	VD
BA_mean_	1	− 0.643	− 0.257*	− 0.111*	− 0.224*	− 0.102*	− 0.107*	− 0.164*
MD_mean_		1	0.259*	0.088*	0.181*	0.144*	0.160*	0.167*
FD			1	0.324*	0.678	0.596	0.634	0.724
*τ*_mean_				1	0.340*	0.147*	0.162*	0.303*
*D*					1	0.284*	0.316*	0.511
NB						1	0.956	0.693
NV							1	0.684
VD								1

Negative correlation coefficient indicates an inverse correlation

*HDMI* high-definition microvasculature imaging

*Correlation coefficient smaller than 0.45 indicates a low-correlation relationship

**Fig. 5:**
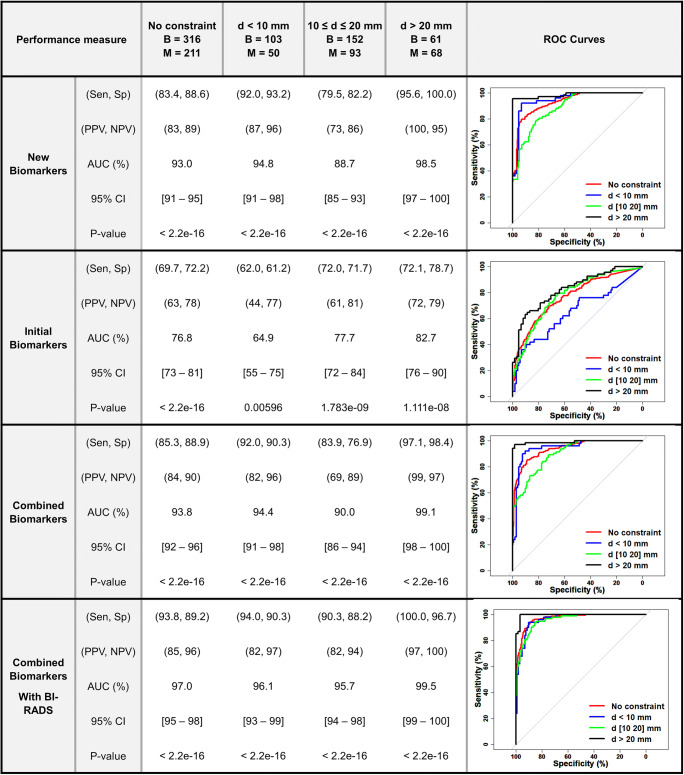
Summary of logistic regression multivariable analysis and ROC curves for differentiating between benign and malignant in different size groups using new/initial HDMI biomarkers and combined new/initial ± BI-RADS. (B, benign; M, malignant; Sen, sensitivity; Sp, specificity; PPV, positive predictive value; NPV, negative predictive value; AUC, area under curve; CI, confidence interval; BI-RADS, Breast Imaging Reporting and Data System. The numbers for Sen, Sp, PPV, NPV, AUC, and CI are given in percentile.) ROC curves for New HDMI Biomarkers, Initial HDMI Biomarkers, Combined HDMI Biomarkers, Combined Biomarkers With BI-RADS

The summary of logistic regression multivariable analysis results in differentiating benign from malignant groups for the new and initial HDMI biomarkers alone as well as the combination of new and initial are shown in Fig. 5. New HDMI biomarkers outperformed the initial biomarkers for classification of breast masses in all lesions regardless of size, showing an AUC of 93.0% (95% CI 91–95%) with sensitivity and specificity of 83.4% and 88.6%, respectively. The best performance was observed in the group of lesions larger than 20 mm, showing an AUC of 98.5% (95% CI: 97–100%), sensitivity of 95.6%, and specificity of 100.0%. The AUC ranged from 93 to 99% depending on the size constraints. Figure 5 also includes the ROC curves for all lesions in different size constraints using new and initial HDMI biomarkers alone and combined. A similar multivariable logistic regression analysis was done by including the BI-RADS score [35] as an additional parameter, showing an AUC, sensitivity, and specificity of 97% (95% CI: 95–98%), 93.8%, and 89.2%, respectively, for all lesions regardless of mass size. The best classification was achieved in lesions larger than 20 mm, showing an AUC, sensitivity, and specificity of 99.5% (95% CI: 99–100%), 100%, and 96.7%, respectively.

**Fig. 6:**
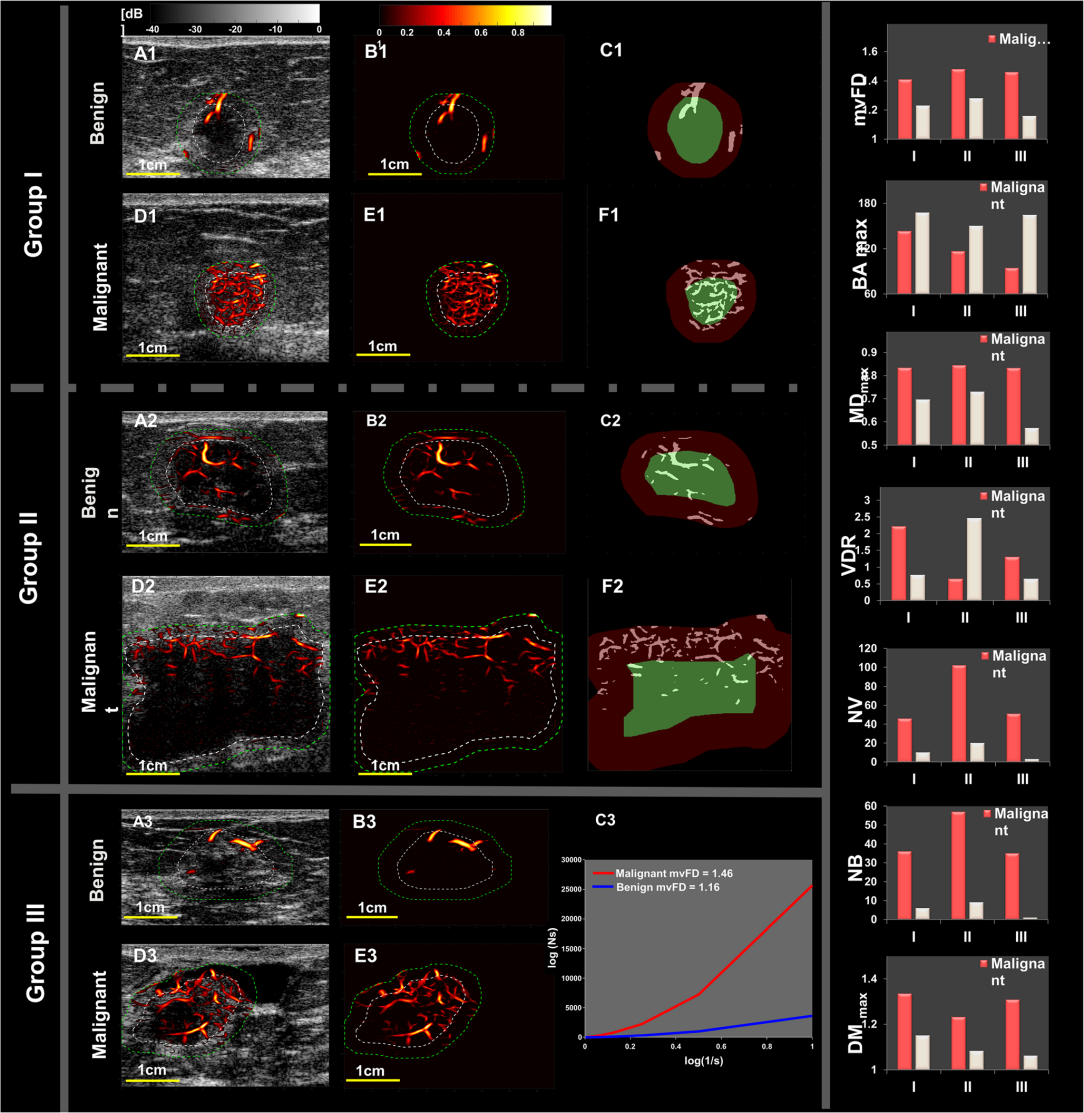
Representative benign and malignant cases: Groups I and II represent SVP diagrams for microvessel images of breast masses. Group III represents mvFD graphs for the microvessel images of a benign and a malignant breast mass. Group I shows breast masses < 10 mm (benign, top row, and a malignant mass, second row). Panels A1, D1, B1, and E1 are the HDMI images. Panels C1 and F1 show the SVP diagrams, indicating peripherally located vessels in the benign mass and centrally located microvessels in the small malignant mass. Group II shows l breast masses > 10 mm (fibroadenoma, top of row, and invasive poorly differentiated ductal carcinoma, NG Gr. III, at the bottom). Panels A2, D2, B2, and E2 are the HDMI images of group II masses. Panels C2 and F2 are the SVP diagrams showing centrally concentrated vessels in the large benign breast mass and peripherally distributed microvessels in the large malignant mass. Group III shows a benign mass (hyalinized fibroadenoma) and a malignant mass (invasive ductal carcinoma, grade II). Panels A3, D3, B3, and E3 are the HDMI images. Panel C3 is the mvFD graph indicating the complexity of microvessels in these two masses. This graph shows a remarkable difference in the complexity between the malignant and benign masses. The bar graphs on the right side of Fig. 6 show that the value of each of the new HDMI biomarkers (mvFD, BA, MD, and VDR (representing SVP)) is remarkably different between the benign and malignant masses in each of the three groups

We have shown six representative cases in 3 pair images of benign and malignant breast masses with the values of new biomarkers in a bar graph and comparative images in Fig. 6. These results are detailed in supplementary materials available online. The combined HDMI biomarkers (initial and new) were also tested on two major benign and malignant histological types with the highest sample size in our study, fibroadenoma (*n* = 114) and invasive ductal carcinoma (*n* = 138). The ROC analysis resulted in an area under the ROC curve of 97.1%, a sensitivity of 95.5%, and a specificity of 98.7% (Figure B-Suppl; please see Supplementary material, available online).

## Discussion

This study investigated the performance of the novel quantitative biomarkers of contrast-free high-definition microvessel imaging (HDMI) for differentiating malignant and benign breast masses. Our findings show that four new HDMI biomarkers, SVP calculated by VDR, mvFD, BA, and MD, provided meaningful separation between malignant and benign lesion groups and outperformed our initial biomarkers (vessel diameter, vessel density, tortuosity, number of vessel segments, and number of branch points) [24]. The multivariable analysis using a logistic regression classification method with all new biomarkers provided consistently better discrimination performance than any individual biomarker alone. Additionally, the discrimination power improved as tumors grow. The addition of BI-RADS scores based on US descriptors to the multivariable analysis using all biomarkers remarkably increased the sensitivity, specificity, and AUC in all size groups. This finding suggests that new quantitative HDMI offers complementary diagnostic information to conventional ultrasound for increased accuracy in breast cancer diagnosis. Moreover, an important advantage of this new tool is that it does not require injection of a contrast agent for better vessel enhancement. The envisioned strategy for the clinical use of the quantitative HDMI technique includes the following steps: (1) the ultrasound machine with the associated HDMI processing technique automatically processes the data providing the quantitative biomarkers. (2) The quantitative HDMI biomarkers will be further input to the prediction model implemented in the ultrasound machine to calculate the malignancy probability. (3) Then, the radiologist reads the malignancy probability and compares the value with the threshold for decision-making. The clinical application value of this HDMI technology is as follows: (1) if the malignancy probability calculated with the prediction equation is lower than the threshold, the algorithm would be more supportive of follow-up. As such, this model could help clinical decision-making, possibly downgrading a presumptive BI-RADS 4a lesion to a BI-RADS 3 with recommendation for follow-up. (2) If the malignancy probability is higher than the threshold, the algorithm would be supportive of breast biopsy. With additional validation, refinement, and testing with multi-center large-population studies, the threshold would be further validated.

Few studies have proposed ultrasound microvessel imaging for differentiation of breast masses, either with [19, 22] or without [37] contrast agents, with limited patient studies using a few morphological biomarkers. The current quantitative HDMI study includes a wide range of tumor microvessel morphological biomarkers tested on a relatively large group of patients. An additional advantage is that the enhancement and visualization of tumor vessels at the submillimeter level can be done without the need for contrast agents. Moreover, our method is capable of quantifying vessel diameter, which may be challenging in contrast-enhanced tracking approaches [38].

This research investigates the performance of MD, BA, mvFD, and SVP as new morphological biomarkers of tumor microvessels in contrast-free ultrasound microvessel imaging for differentiation of breast lesions. The diagnostic value of MD was demonstrated for different diseases [39–42], indicating that the vascular network of diseased tissue could show a deviation from Murray’s law [43]. Our study also showed a higher MD in malignant breast lesions. Moreover, our study found a statistically significant decrease in BA in malignant breast lesions. Similarly, a decreased BA in invasive carcinomas of the colon has been shown in a previous study [30]. In our study, mvFD was found to have higher values in malignant compared to benign lesions for all size constraints. This finding is consistent with the results of other studies, indicating that microvascular complexity calculated by mvFD may provide important diagnostic and prognostic information as well as insight into tumor angiogenesis [27, 28]. In our study, the SVP biomarker indicated that peripherally concentrated vascularity in larger tumors (diameter > 20 mm) is associated with malignancy; however, in smaller tumors (diameter ≤ 20 mm), a centrally concentrated vascularity is an indicator of malignancy. This finding is also consistent with other studies suggesting that small malignant tumors have few large vessels in the periphery, but as the tumor enlarges, the vessel density decreases in the central area and the microvessels tend to have more peripheral distribution [4, 44, 45]. If there are no or few microvessels within the lesion, the quantitative HDMI could classify the lesion as benign.

In this study, *D*_max_ was statistically significantly higher in malignant lesions compared to benign masses. In fact, using *D*_max_, one can test the possibility of a major feeding vessel that may be indicative of malignancy. This result is consistent with the fact that VEGF-A forms numerous larger blood vessels (presumably mother vessels) in the periphery of malignant tumors, but fewer and smaller vessels in the central part of the tumor [4, 44, 46]. Therefore, maximum vessel diameter has a better discriminatory power than averaging the diameter of the vessels. A similar observation was also made for vessel tortuosity. With tortuosity averaged over the entire vascular bed, there were no statistically significant differences between malignant and benign in all size groups; however, the maximum vessel tortuosity was statistically significantly higher in the malignant cases compared to the benign cases. These findings concur with the fact that, as a malignant tumor enlarges, more tortuous vessels with increased diameter are seen at the tumor–host interface than in the central region [4], indicating that averaging these biomarkers has less diagnostic value than determining their maxima. This indicates that vessel tortuosity analysis can offer information complementary to flow imaging and may offer additive value in discrimination when both benign and malignant tumors are hypervascular [47]. The increased numbers of branch points and vessel segments in our study signify a greater level of vessel sprouting, endorsing them as discriminators of benign and malignant tumors [48].

Additionally, HDMI biomarkers were statistically significantly different between higher and lower NG grades of malignant breast tumors. Higher values of mvFD, a marker of vessel complexity, NB, NV, and VD were seen in the higher grades of breast carcinomas. Previous studies reported a higher microvessel density, sprouting, and structural irregularity associated with higher pathological grades of breast carcinomas that may lead to higher incidences of metastasis and a poorer prognosis [28, 49, 50].

One limitation in this study is that the quantitative biomarkers were estimated using 2D HDMI which may overlook some important 3-dimensional (3D) morphological features and the connectivity of tumor microvessels, potentially leading to either underestimation or overestimation of these features. To address these limitations, a complementary study would involve quantitative 3D HDMI imaging and morphometric analysis using either a mechanical scanning system equipped with a linear array [51] or a matrix ultrasound transducer [52] for volumetric imaging. Such approaches would enable a more comprehensive vessel morphological analysis. To keep a single gold reference standard for all patients, the pathology results of core-needle biopsy rather than the surgical pathology served as the gold reference standard. As surgical pathology is not available in benign lesions that do not normally have surgical excision for treatment and in the group of complete pathological responders to neoadjuvant therapy will be no cancer. However, the histological features of cancer in core needle biopsy were the same as with surgical pathology.

Future work should also focus on using the emerging radiomic analysis approach by incorporating a data characterization algorithm to extract numerous features from images. Although radiomic analysis has its own challenges [53], it may have the potential to facilitate improved clinical decision-making [54]. Another direction for improving diagnostic performance of ultrasound is to combine our microvasculature morphometric analysis with established conventional ultrasound metrics. Conventional ultrasound provides information about the shape and texture of a breast lesion to aid in cancer detection, while our quantitative microvasculature method provides information related to angiogenesis. Combining these two pieces of information may improve the overall diagnostic performance of ultrasound. The HDMI study was performed on patients with suspicious breast lesions detected by clinical ultrasound and scheduled for biopsy. Nearly all cases were classified as BI-RADS 4 and 5. Therefore, we believe it would not be fair to compare the sensitivity and specificity of our method to conventional ultrasound since limited numbers of BI-RADS categories lower than 4 were included in our study. A future study could include microvasculature morphometric analysis of breast lesions regardless of their BI-RADS category, with the caveat that cases in lower BI-RADS categories will not have pathology results for comparison. The focus of the present study is to validate the performance of the new HDMI biomarkers for breast lesion differentiation on a large patient population. For future studies, we would like to compare the performance of HDMI to other diagnostic methods, e.g., B-mode, color Doppler, and the contrast-enhanced ultrasound.

In conclusion, the efficacy of the four novel quantitative biomarkers of the HDMI method for breast cancer detection is promising. The fact that HDMI does not require injection of a contrast agent simplifies its use in routine clinical practice. In the future, the proposed method with new biomarkers can offer a new means of detecting breast cancer when used as a complementary imaging tool to conventional ultrasound.

## Supplementary Information

ESM 1 (DOCX 289 kb)

## Abbreviations

AUC: Area under the curve
BA: Bifurcation angle
CI: Confidence interval
DM: Distance metric
HDMI: High-definition microvasculature imaging
IDC: Invasive ductal carcinoma
mvFD: Microvessel fractal dimension
NB: Number of branch points
NG: Nottingham
NV: Number of vessel segments
ROC: Receiver operating characteristics
SVP: Spatial vascularity pattern
VD: Vessel density
VEGF: Vascular endothelial growth factors

## Declarations

### Guarantor

The scientific guarantors of this publication are Dr. Azra Alizad (Department of Radiology) and Dr. Mostafa Fatemi (Department of Physiology and Biomedical Engineering), Mayo Clinic.

### Statistics and biometry

Dr. Eric C. Polley kindly provided statistical advice for this study and was listed as one of the authors.

### Study subjects or cohorts overlap

A small subset of subjects have been previously reported in the following article [1]. In the prior published study, we introduced the basic principles of 4 new quantitative biomarkers based on microvessel images including some simulation and limited patient study results to illustrate the role of each biomarker. The four biomarkers are (1) microvessel fractal dimension, (2) Murray’s deviation, (3) bifurcation angle (BA), and (4) spatial vascular pattern. In the present study, we introduce the processing methods for the new high-definition microvessel imaging technique and quantify comprehensive microvessel morphological parameters of breast tumor, then validate the method on a large group of patient population. The present study with a larger sample size and more lesion categories allowed us to investigate the performance of HDMI biomarkers in different lesion size groups individually and together in a multivariable analysis. Furthermore, the correlation of HDMI biomarkers with cancer grades has been investigated. Therefore, the current validation study is a substantial expansion in knowledge compared to our previous work.

Ternifi R., Wang Y., Polley E.C., Fazzio R.T., Fatemi M., and Alizad A. (2021) Quantitative biomarkers for cancer detection using contrast-free ultrasound high-definition microvessel imaging: fractal dimension, Murray’s deviation, bifurcation angle & spatial vascularity pattern. IEEE Transactions on Medical Imaging Early Access:1-10

### Methodology

• prospective

• diagnostic or prognostic study

• performed at one institution
