# Supplementary material for: Ultrasound high-definition microvasculature imaging with novel quantitative biomarkers improves breast cancer detection accuracy
Source: Eur Radiol. 2022 Apr 29;32(11):7448–62. doi: 10.1007/s00330-022-08815-2 (PMC9616967; doi:10.1007/s00330-022-08815-2)
Supplement: Supplementary file 1 — (DOCX 289 kb) [file 330_2022_8815_MOESM1_ESM.docx]

**High-definition microvasculature imaging and vessel extraction**

Recently, it has been demonstrated that slow blood flows can be successfully decoupled from strong tissue clutter signal when highly registered spatial-temporal data were processed in the singular-spectrum domain [24;26]. This approach provides tissue-blood echo separation, even in the presence of a strong Fourier spectral overlap, thus preparing the image for quantitative analysis of the microvasculature network. Noting that this method can visualize small sub-millimeter vessels, as small as 300 µm, we have termed this approach high-definition microvasculature imaging (HDMI). This quantitative HDMI approach is based on 3 major steps, 1) image formation by recording a large sequence of images from breast tumor at a high frame rate and followed by a series of vessel filtering to enhance the vessels; 2) a set of processing steps for morphological filtering and segmentation that prepares the image for quantification; and 3) quantification of vessel morphology using the quantification tools. Given the prominent role of angiogenesis in breast cancer resulting in structurally abnormal microvessels, quantitative HDMI may be particularly well suited for differentiation of malignant from benign breast masses.

**Method:** In this study, all imaging acquisitions were performed using an Alpinion Ecube12-R ultrasound machine (ALPINION Medical Systems), and a linear array L3-12H operating at 8.5 MHz. Data was then downloaded from the machine and all processing was performed offline using MATLAB. Clutter signal was removed using the singular value thresholding method [23]. The tissue clutter rank was automatically defined based on the decay rate of the singular values as described previously [23]. Background noise normally appears after singular value thresholding and limits microvessel visibility. We used a novel two-step algorithm to decrease severe intensity variations of the Doppler signal and to reduce the background noise. We also used a series of morphology-based vessel filtering for removing the global and local background signal. We then used a multi-scale Hessian-based vessel-enhancement filtering to extract the vascular structures.

**High-definition Microvasculature imaging Quantitative Biomarkers**

Definitions and calculations of the new [25] and initial biomarkers[24] are detailed in Table 1. Four new HDMI biomarkers are briefly described here: 1) Microvessel fractal dimension (mvFD) is a marker of structural complexity of tumor microvessels that can provide additional diagnostic and prognostic information [25; 27; 28]. 2) Bifurcation angle (BA) refers to the angle between two daughter vessels. Studies report a decrease of BA in malignant tumors [25; 29; 30]. 3) Murray’s deviation (MD) is a diameter mismatch when daughter vessels are wider than predicted by Murray's law. Increased MD is seen in the vasculature network of malignant tumors [25; 31; 32]. 4) Spatial vascularity pattern (SVP) describes the distribution pattern of vascular networks [25; 33]. The SVP was calculated by the vessel density ratio (VDR) between the segmented center and peripheral regions of the lesion obtained by the image erosion operator [34]. The discriminatory definition is based on breast mass size. In breast masses 20mm or smaller, peripherally concentrated vascularity (VDR<1) or SVP=0 is associated with benign diagnosis, however, in breast masses larger than 20mm it is associated with malignant diagnosis. In breast masses larger than 20mm, centrally concentrated vascularity (VDR>1) or SVP=1 is associated with benign diagnosis, however, in breast masses 20mm or smaller it is associated with malignant diagnosis. We have also extracted the initial biomarkers described in [24].

**Method for Microvessel morphometric analysis**

For each lesion, a quantification region of interest (ROI) was defined based on the lesion boundaries acquired from the B-mode ultrasound images. The initial ROI was dilated 2mm to include peripheral vascularity. After image formation, a set of processing steps will be performed to prepare the image for quantification. In a previous study, we presented the methods for quantification of vessel morphological parameters in a contrast-free microvessel imaging [124]. As shown in Figure A, vessel segmentation includes the following steps: converting the microvasculature image (output of Hessian filter) to a binary image, removing small noise-like objects through an erosion and dilation operation, determining the mid-line of each vessel and constructing the full skeleton of the vessel network [23-25]. Finding the skeleton is based on a thinning algorithm [24; 55]. In this approach, vessels are sequentially thinned, and the midline of each vessel determined to construct the skeleton of a vessel network.


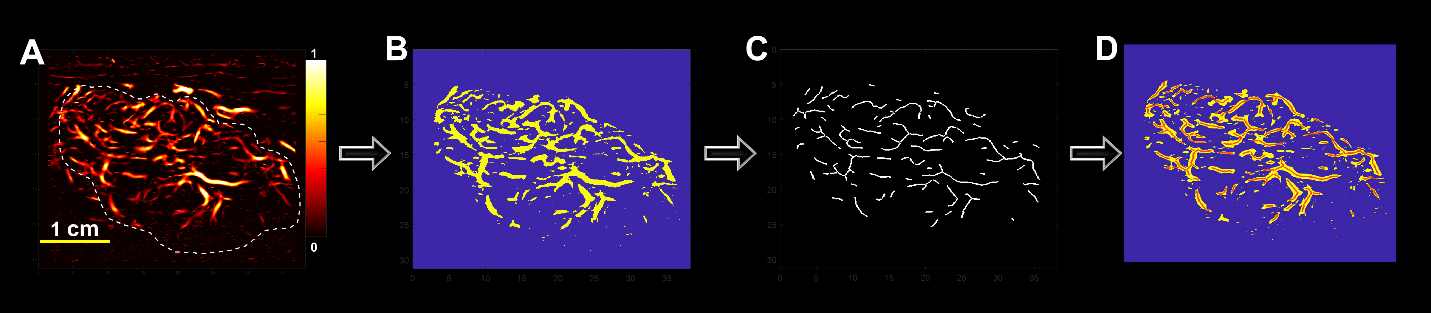
**Figure A:** A set of processing steps after image formation and segmentation to prepare the image for quantification: (A) microvasculature image of malignant breast tumor, (B) conversion of the microvasculature image into a binary image, (C) skeleton showing the network of vessels, (D) overlaid of the skeleton on the binary vessel image with identified branching points.

After these steps, the output image includes the vessel segments. These vessel segments were analyzed to estimate the desired quantitative morphological parameters of the vessels. A series of new parameters including microvessel fractal dimension (mvFD), Spatial vascularity pattern (SVP), Bifurcation angle (BA), Murry’s deviation (MD) [25], as well as our previously described parameters in [24] including, number of vessel segments (NV), vessel density (VD), number of branch points (NB), vessel diameter (D), and vessel tortuosity and vessel diameter were analyzed.

**Clinical pathologic data**

The expressions of immunohistochemical (IHC) biomarkers were obtained from core needle biopsies for the malignant lesions reported in clinical reports, including estrogen receptor (ER), progesterone receptor (PR), human epidermal growth factor receptor 2 (HER2), and Ki-67 proliferation index. Reporting criteria for ER and PR status was based on <1% reactive cells were negative and ≥1% reactive cells were positive. HER2 score 0, 1+ were considered as negative and score of 3+ was considered as positive. Fluorescence in situ hybridization (FISH) report was checked if HER2 score was 2+. Positive FISH amplification was considered as HER2 positive. Based on the four IHC biomarkers, the malignant lesions were divided into five molecular subtypes according to theSt. Gallen criteria [56]. Luminal A includes lesions with ER positive, PR positive/negative, HER2 negative, Ki-67<14%. Luminal B with HER2+ includes lesions with ER positive, PR positive/negative, HER2 negative, and Ki-67≥14%. Luminal B with HER2- includes lesions with ER positive, PR positive/negative, HER2 positive, any Ki-67. HER2+ includes lesions with ER negative and PR negative, HER2 positive. Triple-negative (TN) includes lesions with ER negative, PR negative and HER2 negative.

**Results of Figure 6**

Figure 6 shows HDMI images of six representative cases in 3 groups labeled I, II, and III.  Each group includes one benign and one malignant lesion. The bar graphs on the right side of Figure 6 show the values of the new HDMI biomarkers, SVP (calculated by VDR), mvFD, BA and MD for each benign-malignant pair in each group. Compared to benign cases, the malignant cases show increased mvFD and MD, but decreased BA in all groups, as expected. The SVP diagrams for group I with small lesions (panels C1 and F1) clearly show peripherally concentrated microvessels in the benign mass but centrally concentrated microvessels in the malignant mass. Conversely, the SVP diagram in group II with large lesions (panels C2 and F2) clearly shows centrally concentrated microvessels in the benign mass but peripherally concentrated microvessels in the malignant mass. In group III, the graph shows a remarkable difference between the mvFD estimates of the malignant and benign breast masses. The values of the new biomarkers and three of the initial biomarkers (NV, NB, and DM_max_) are considerably higher in the malignant cases than in benign tumors for all three groups (Figure 6).

**Performance of HDMI in differentiating fibroadenoma from invasive ductal carcinoma:** The combined HDMI biomarkers (initial and new) were also tested on two major benign and malignant histological types with the highest sample size in our study, fibroadenoma (n=114) and invasive ductal carcinoma (n=138). The ROC analysis resulted an area under ROC curve of 97.1%, a sensitivity of 95.5% and a specificity of 98.7% (Figure B).”


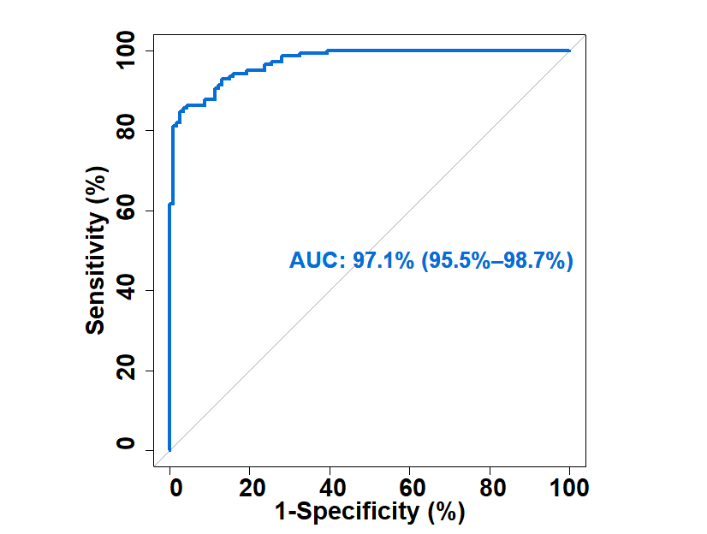


**Figure B. ROC curve generated with the combined new and initial HDMI biomarkers and BI-RADS score for differentiating Fibroadenoma from invasive ductal carcinoma.**

**References**

55. Goldhirsch A, Wood WC, Coates AS et al (2011) Strategies for subtypes—dealing with the diversity of breast cancer: highlights of the St Gallen International Expert Consensus on the Primary Therapy of Early Breast Cancer 2011. Annals of oncology 22:1736-1747

56 Demené C, Deffieux T, Pernot M et al (2015) Spatiotemporal clutter filtering of ultrafast ultrasound data highly increases Doppler and fUltrasound sensitivity. IEEE transactions on medical imaging 34:2271-2285

57 Lam L, Lee S-W, Suen CY (1992) Thinning methodologies-a comprehensive survey. IEEE Transactions on pattern analysis and machine intelligence 14:869-885

**Supplemental table**

Summary of Classification Performance Based on Different Vessel Morphological Parameters for Differentiating between Benign and Malignant Groups Considering Lesion Size.

| Mass size (mm) | Evaluation | mvFD | VDR | MD | MD_max_ | BA | BA_max_ | VD | NV | NB | D | D_max_ | DM | DM_max_ |
| --- | --- | --- | --- | --- | --- | --- | --- | --- | --- | --- | --- | --- | --- | --- |
| All  B=316  M=211 |  |  |  |  |  |  |  |  |  |  |  |  |  |  |
|  | p-value | <.001 | <.001 | <.001 | <.001 | <.001 | <.001 | <.001 | <.001 | <.001 | <.001 | <.001 | <.001 | <.001 |
|  | Sen(%) | 71% | 76 | 80 | 70 | 86 | 86 | 67 | 70 | 63 | 75 | 65 | 69 | 72 |
|  | Sp(%) | 70% | 75 | 72 | 72 | 68 | 61 | 63 | 68 | 68 | 46 | 66 | 56 | 67 |
|  | PPV(%) | 78 | 76 | 85 | 78 | 60 | 45 | 70 | 73 | 64 | 74 | 69 | 73 | 78 |
|  | NPV(%) | 61 | 76 | 66 | 63 | 41 | 36 | 60 | 64 | 69 | 48 | 61 | 51 | 59 |
|  | AUC (%) | 74 | 77 | 83 | 73 | 80 | 75 | 66 | 76 | 72 | 60 | 66 | 62 | 71 |
|  | 95% CI | [70-79] | [72-82] | [79-86] | [68-77] | [75-84] | [71-80] | [70-79] | [71-80] | [66-77] | [55-64] | [61-71] | [57-67] | [66-75] |
| d<10  B=103  M=50 |  |  |  |  |  |  |  |  |  |  |  |  |  |  |
|  | p-value | <.001 | <.001 | <.001 | <.001 | <.001 | <.001 | .003 | <.001 | .003 | .76 | 0.17 | .40 | <.001 |
|  | Sen(%) | 70 | 84 | 78 | 76 | 80 | 84 | 57 | 62 | 66 | 64 | 62 | 63 | 56 |
|  | Sp(%) | 65 | 81 | 76 | 74 | 80 | 70 | 74 | 72 | 62 | 42 | 80 | 72 | 71 |
|  | PPV(%) | 82 | 88 | 88 | 86 | 44 | 70 | 76 | 78 | 71 | 71 | 74 | 40 | 77 |
|  | NPV(%) | 49 | 76 | 61 | 59 | 59 | 72 | 54 | 55 | 56 | 35 | 56 | 54 | 49 |
|  | AUC (%) | 68 | 86 | 86 | 82 | 82 | 75 | 67 | 73 | 68 | 52 | 69 | 63 | 72 |
|  | 95% CI | [58-77] | [78-93] | [80-92] | [74-90] | [73-90] | [65-85] | [55-77] | [63-82] | [57-80] | [42-61] | [49-79] | [52-74] | [52-82] |
| 10≤d≤20  B=152  M=93 |  |  |  |  |  |  |  |  |  |  |  |  |  |  |
|  | p-value | <.001 | <.001 | <.001 | <.001 | <.001 | <.001 | <.001 | <.001 | <.001 | .003 | .002 | <.001 | <.001 |
|  | Sen(%) | 68 | 68 | 80 | 71 | 84 | 80 | 57 | 77 | 67 | 78 | 58 | 70 | 68 |
|  | Sp(%) | 74 | 79 | 73 | 86 | 71 | 68 | 72 | 65 | 56 | 68 | 68 | 61 | 71 |
|  | PPV(%) | 80 | 70 | 86 | 78 | 81 | 73 | 68 | 77 | 62 | 78 | 67 | 77 | 78 |
|  | NPV(%) | 61 | 77 | 64 | 72 | 71 | 64 | 62 | 63 | 61 | 48 | 60 | 53 | 59 |
|  | AUC (%) | 76 | 76 | 84 | 70 | 81 | 75 | 66 | 73 | 69 | 61 | 63 | 67 | 73 |
|  | 95% CI | [70-82] | [69-83] | [79-89] | [62-78] | [75-87] | [69-82] | [58-73] | [66-80] | [60-77] | [55-71] | [55-71] | [60-79] | [66-71] |
| d>20  B=61  M=68 |  |  |  |  |  |  |  |  |  |  |  |  |  |  |
|  | p-value | <.001 | <.001 | <.001 | .002 | <.001 | <.001 | .004 | <.001 | <.001 | .06 | <.001 | .15 | .001 |
|  | Sen(%) | 74 | 76 | 78 | 66 | 82 | 90 | 80 | 71 | 82 | 66 | 63 | 71 | 68 |
|  | Sp(%) | 69 | 73 | 67 | 61 | 74 | 50 | 77 | 73 | 60 | 46 | 67 | 46 | 60 |
|  | PPV(%) | 70 | 69 | 73 | 62 | 75 | 64 | 68 | 65 | 68 | 55 | 58 | 58 | 62 |
|  | NPV(%) | 73 | 80 | 73 | 65 | 72 | 59 | 70 | 72 | 76 | 58 | 72 | 60 | 65 |
|  | AUC (%) | 72 | 73 | 76 | 66 | 70 | 73 | 66 | 78 | 72 | 60 | 70 | 57 | 67 |
|  | 95% CI | [62-81] | [62-82] | [68-85] | [57-76] | [60-82] | [64-81] | [54-76] | [69-86] | [61-82] | [50-70] | [61-80] | [47-68] | [50-80] |

Note. - The numbers for Sen, Sp, PPV, NPV, AUC and CI are given in percentile. d is breast mass size. B = benign, M = malignant, Sen = sensitivity, Sp = specificity, PPV = positive predictive value, NPV = negative predictive value, AUC = area under curve, CI = confidence interval.
